# Sedentary behavior and altered metabolic activity by AgNPs ingestion in *Drosophila melanogaster*

**DOI:** 10.1038/s41598-017-15645-6

**Published:** 2017-11-15

**Authors:** Akanksha Raj, Prasanna Shah, Namita Agrawal

**Affiliations:** 1 Department of Zoology, University of Delhi, Delhi, 110007 India; 2 Acropolis Institute of Technology and Research, Indore, 453771 India

## Abstract

Among several nanoparticles, silver nanoparticles (AgNPs) are extensively used in a wide variety of consumer products due to its unique antimicrobial property. However, dosage effect of AgNPs on behavior and metabolic activity in an *in vivo* condition is not well studied. Therefore, to elucidate the impact of AgNPs on behavior and metabolism, systematic and detailed dosages study of AgNPs was performed by rearing *Drosophila melanogaster* on food without and with AgNPs. We found that dietary intake of AgNPs at early larval stage leads to behavioral abnormalities such as poor crawling and climbing ability of larvae and adults respectively. Interestingly, intake of higher dosage of AgNPs at larval stage significantly altered metabolic activity that includes lipid, carbohydrate and protein levels in adult flies. Further, detailed analysis revealed that AgNPs causes remarkable reduction in the number of lipid droplets (LDs) which are lipid storage organelles in *Drosophila*. We also observed an increased production of reactive oxygen species (ROS) in AgNPs ingested larval tissues. These results strongly imply that higher dosage of AgNPs ingestion from early larval stage of *Drosophila* is inimical and thereby draws concern towards the usage of AgNPs in consumer goods.

## Introduction

The innovative concept of nanotechnology for precisely designing the nano-sized particles has been incredibly beneficial in improving the quality of consumer goods^1^. The modern science of nanotechnology offers a wide range of engineered nanoparticles among which silver nanoparticles (AgNPs) have gained significant attention due to its extraordinary anti-microbial property^2^. With a better understanding of properties related to nanoparticles, AgNPs are being integrated in various consumer goods^3^ such as cosmetics^4,5^, disinfectant products, biomedical devices^6,7^, wound dressing^8–10^, food packaging, clothing and textiles^11^. Human exposure to nanoparticles may occur through inhalation, dermal contact, and ingestion^12–16^. Therefore, increasing usage and exposure to AgNPs containing consumer products such as cosmetics and various household products demand for a systematic evaluation of its impact on human health.

Reports on AgNPs mediated toxicity has been linked to different aspects such as delay in developmental growth and time, poor rate of survival, decreased fecundity, reduction in body weight and poor climbing^17–19^, but it’s *in vivo* impact on metabolic aspect remains largely unknown at this stage.

*Drosophila* represents a very promising model system to answer several issues related to human health as various physiological functions including the basic metabolic functions remain conserved between the two^20–24^. The impact of AgNPs on lipid droplet accumulation has been recently studied using *Drosophila* as a model system. Accumulation of glial lipid droplets has been reported to elevate ROS level and neuronal mitochondrial defects which promotes neurodegeneration in *Drosophila* ^25^.

An extensive physico-chemical characterization of commercially purchased AgNPs used in the present study was conducted and reported earlier^26^. A significant reduction in body size, loss in body weight and altered behavior of flies eclosed from larvae fed with the higher dosage of AgNPs led us to speculate that the reason behind it could be either less food intake or altered energy reservoir or combination of both. Therefore, in an attempt to find out the possible reason contributing to smaller body size, reduced body weight and behavioral abnormalities in *Drosophila*, we aimed towards quantitation of food intake and various metabolic components such as lipid, carbohydrates, and protein levels of the progeny fed with food-supplemented with different doses of AgNPs. Additionally, the production of higher level of ROS and oxidative stress has been reported to be the predominant mechanism leading to nanotoxicity^27–30^. Therefore, we further investigated if AgNPs causes any alteration in the metabolic activity and ROS production in *Drosophila*. We found that flies have reduced food intake, alteration of metabolic components and significant increase in ROS levels at higher concentration of AgNPs.

## Results

### Dietary intake of AgNPs at larval stage mediates behavioral abnormalities

**Figure 1:**
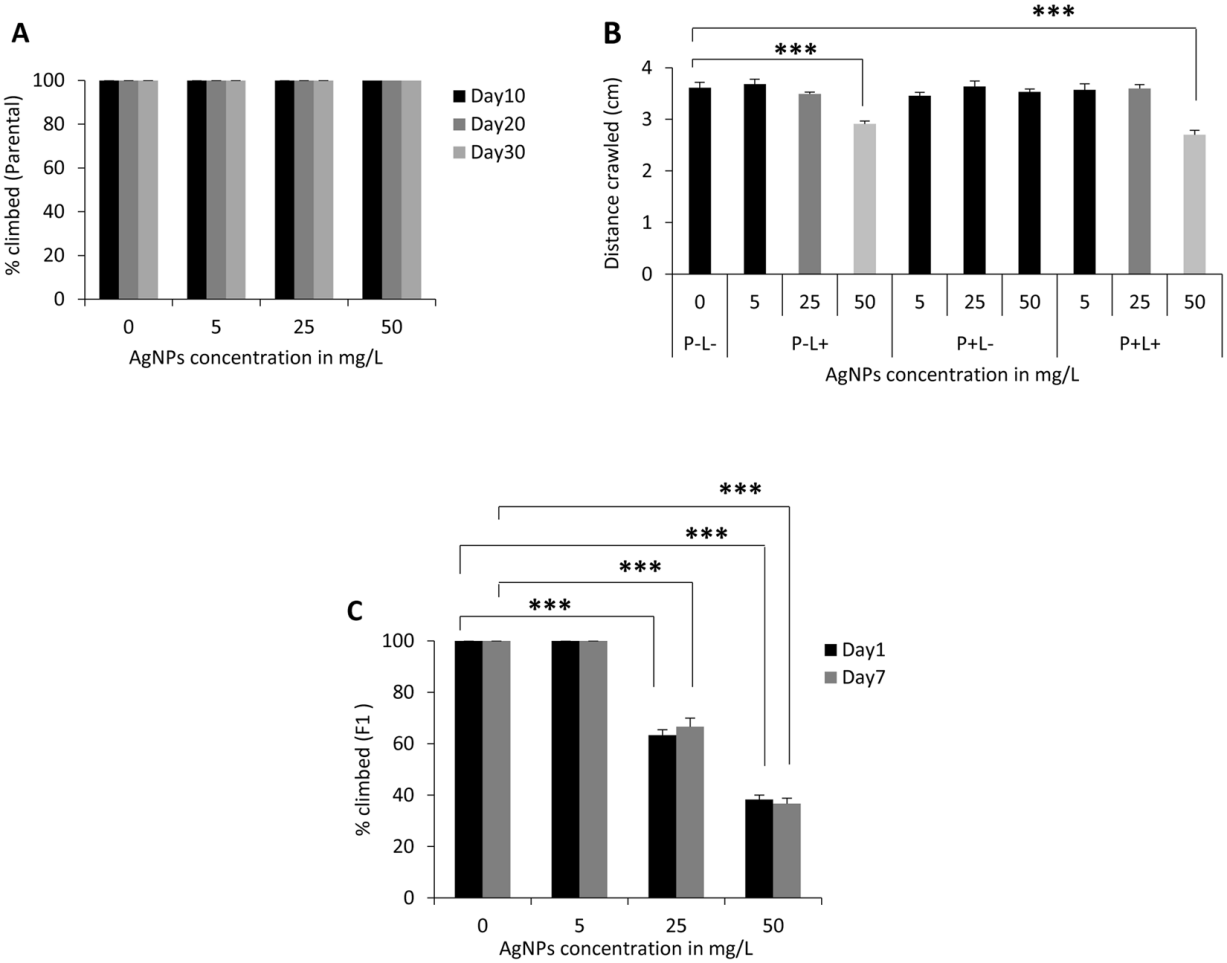
Compromised behavioral activity of F1 progeny reared on AgNPs-supplemented food. (**A**) No effect on vertical climbing of AgNPs-supplemented and not supplemented (control) adult flies aged for short 10 and long 20 and 30 days. (**B**) Dose-dependent impairment in crawling ability of wandering F1 third instar larvae eclosed from different treatment conditions (P − L−/P − L + /P + L−/P + L+). (**C**) Significant reduction in climbing behavior of F1 adult progeny at an age of day 1 and 7 reared on 25 and 50 mg/L of AgNPs (Please note that P + L− climbing data was comparable to P − L− and P − L+ was comparable to P + L+; therefore only P− L− (0 mg/L) and P − L+ data is shown in the graph). Significance was calculated by using an analysis of variance (ANOVA) followed by Tukey-Kramer MSD post hoc test (MSD α_0.05_: Crawling (F1) = 0.84; Climbing (F1) = 28.4). ***p < 0.001 (Student’s t-test).

Different doses of AgNPs-supplemented food were administered at adult (parental) and early larval stage (F1) to evaluate its impact at different developmental stages on behavior. As a measure of behavioral activity for prolonged AgNPs ingestion, we monitored climbing ability of flies aged for 10, 20 and 30 days on food-supplemented with different doses of AgNPs (Fig. 1A). Interestingly, we did not find negative impact of any dose of AgNPs at all the time points on climbing ability as compared to age matched non-fed control flies.

Since adults (parental) were not affected by AgNPs ingestion, we investigated the effect of AgNPs by rearing their F1 progeny from early first instar larvae on food-supplemented without (control) and with different doses of AgNPs. Therefore, eggs laid by parental adults were grown on four different treatment conditions (P − L− (control) and P − L+, P + L− and P + L+). The details of these treatment conditions are mentioned in the materials and methods section. The F1 larvae and adult flies hatching from different treatment conditions were monitored for behavioral assays such as larval crawling and adult climbing. Ingestion of AgNPs at early larval stage (P − L+; P + L+) causes significant alteration in crawling and climbing behavior in a dose-dependent manner (Fig. 1B and C). Moreover, we have earlier reported flight deficit in these F1 adult progeny eclosing from larvae fed with higher dose of AgNPs^31^. It is noteworthy that F1 larvae fed with a dose higher than 50 mg/L had significant effect on survival^26^ and therefore, we could perform behavioral assays of F1 adult flies only up to a dose of 50 mg/L.

Our results clearly suggest that ingestion of AgNPs early during development affects their behavior in a dose-dependent manner, however, ingestion during adult stage even for a prolonged time has no effect. Moreover, comparable behavioral dysfunction was observed when AgNPs was ingested at larval stage (P − L+ and P + L+) irrespective of their parents fed on AgNPs-supplemented diet or not.

### Dose-dependent effect of AgNPs ingestion on body color, size and weight

**Figure 2:**
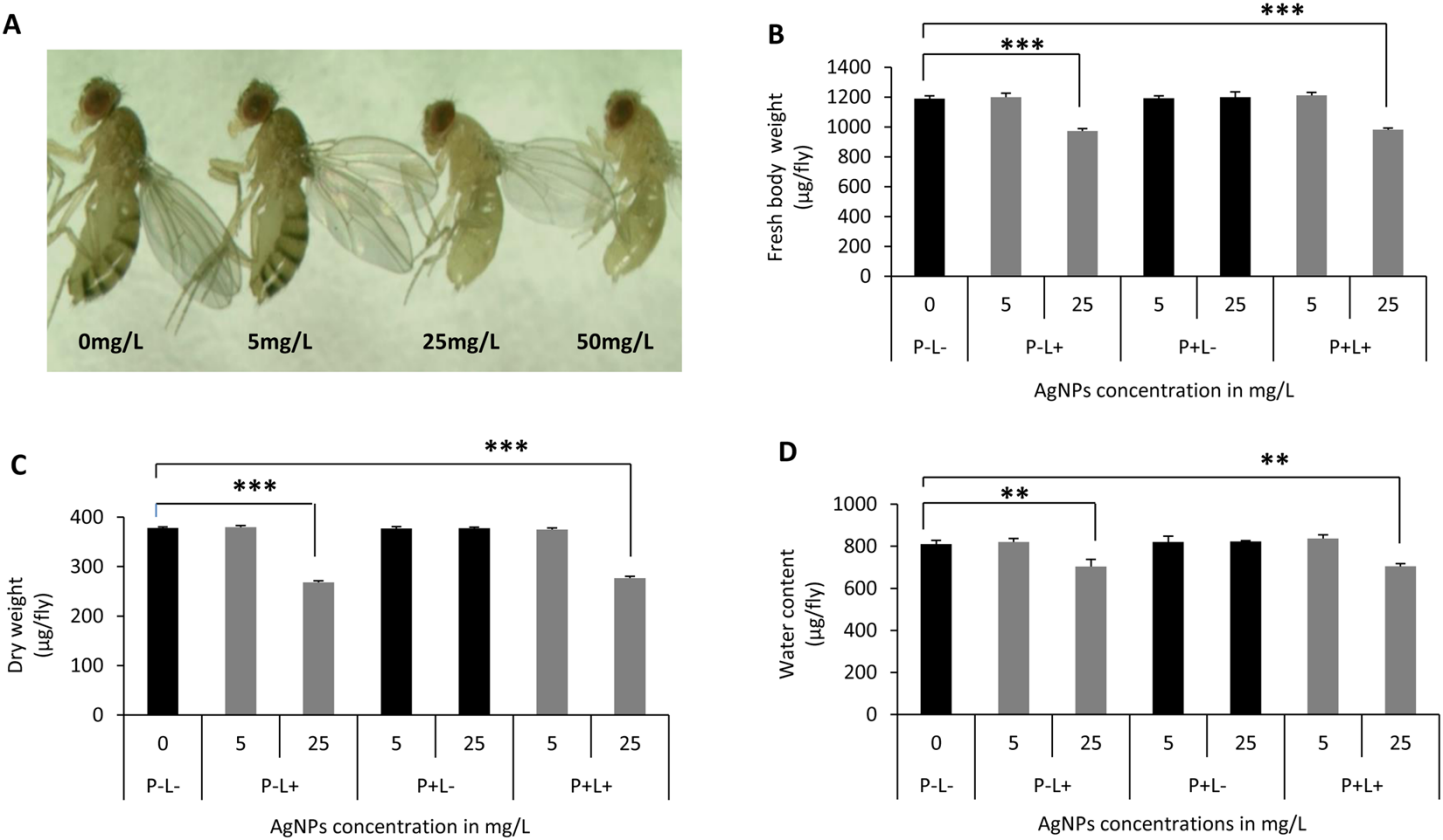
AgNPs ingestion at larval stage causes depigmentation and drop in body weight. (**A**) notable loss in body pigmentation was seen in F1 flies eclosed from larvae reared on AgNPs-supplemented food. Reduction in (**B**) fresh body weight, (**C**) dry weight and (**D**) water content of F1 flies eclosed from all experimental conditions. Significance was calculated by using an analysis of variance (ANOVA) followed by Tukey-Kramer MSD post hoc test (MSD α_0.05_: Fresh weight = 163.4; Dry weight = 41.4; Water content = 227.8). ***p < 0.001 (Student’s t-test).

As reported earlier, we also observed a remarkable loss in body pigmentation^17–19^ and size of flies eclosing from higher dose of AgNPs-supplemented food (Fig. 2A). These observations suggest that larval intake of higher dose of AgNPs causes phenotypic alteration.

Additionally, the F1 adults hatched from larvae fed with AgNPs-supplemented food displayed dose-dependent reduction in fresh body weight as compared to age matched control (Fig. 2B). Since, carbohydrates, proteins and lipids form the major fraction of the body weight, therefore, imbalance in the levels of these crucial macromolecules could be one of the contributing factors in weight alteration. In order to understand if the loss in fresh body weight in these flies is due to alteration in various macromolecules such as lipids, circulating sugar (trehalose), carbohydrate, protein and other structural components, we evaluated the dry weight of the F1 adult progeny. Due to significant reduction in the eclosion rate of the flies from the larvae fed on 50 mg/L of AgNPs and requirement of large sample size to quantitate molecules involved in metabolic activity, highest dose of 25 mg/L was considered. Dietary intake of 25 mg/L of AgNPs at early larval stage resulted in decrease in dry weight (Fig. 2C). Further, flies that eclosed from larvae fed with AgNPs-supplemented food (P − L+ and P + L+), were also found to have reduced levels of water content as compared to age matched control (Fig. 2D). These results indicated that ingestion of AgNPs at early larval stage can possibly cause significant reduction in the level of various molecules such as lipids, carbohydrates, proteins and water that are necessary for normal behavior, body size, weight and metabolic function.

### Impaired feeding behavior of larvae and adult by AgNPs ingestion

**Figure 3:**
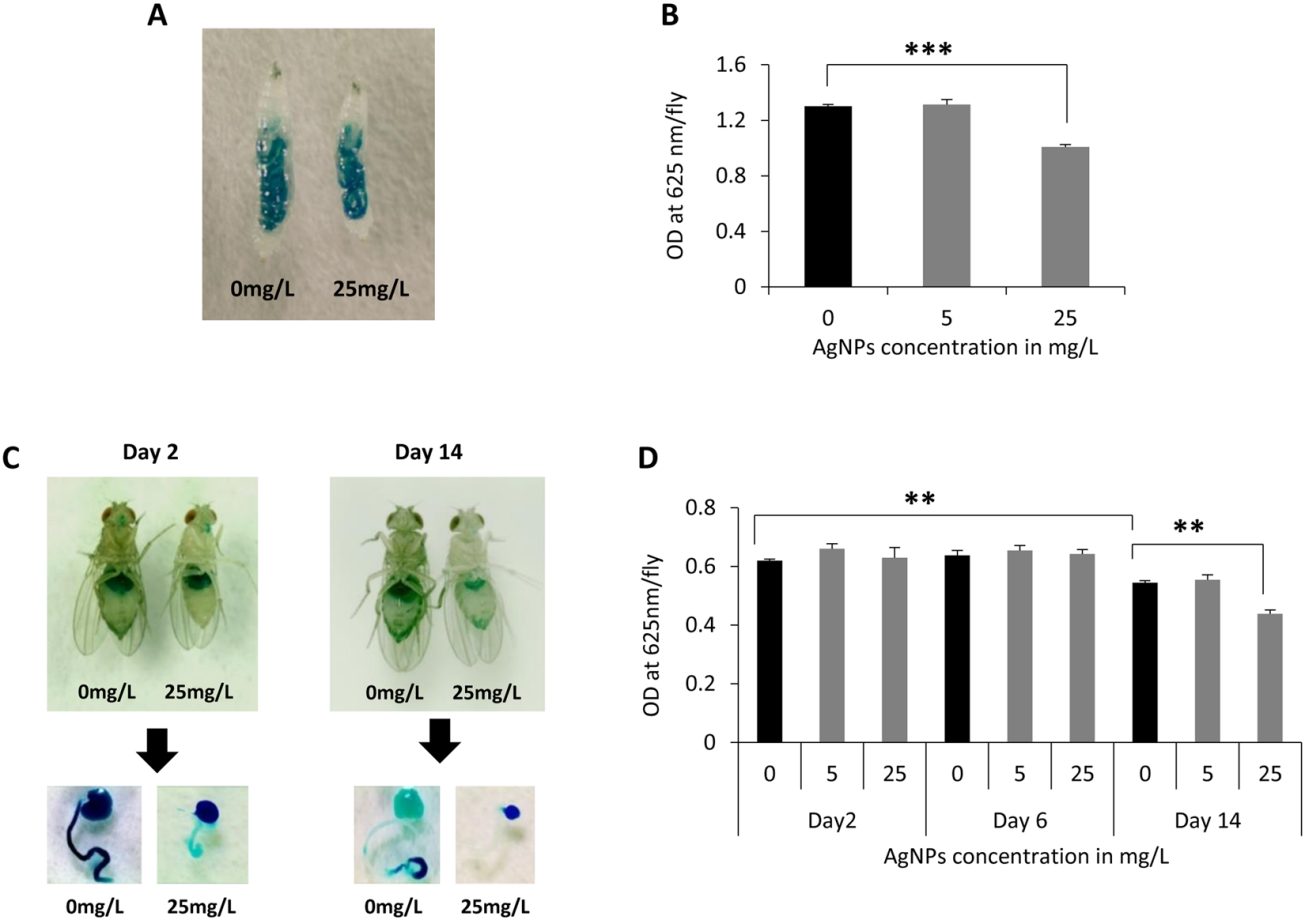
Impedance in feeding behavior upon exposure to AgNPs. (**A**) Larvae reared on standard and AgNPs-supplemented food were fed with a mixture of yeast and 4% blue dye #1. (**B**) Larvae reared on higher dose of AgNPs-supplemented food showed poor rate of feeding as compared to control larvae. (**C**) Adult flies emerged from standard and AgNPs-supplemented food were fed with a mixture of yeast and 2.5% blue dye #1. (**D**) Rate of food intake in F1 adult progeny. Significance was calculated by using an analysis of variance (ANOVA) followed by Tukey-Kramer MSD post hoc test (MSD α_0.05_: Adult feeding = 0.12). ***p < 0.001 (Student’s t-test).

To understand if reduction in body size and weight of adult progeny is also related to food intake, larvae and adults reared on different dosage of AgNPs-supplemented food were fed with the mixture of yeast and blue dye #1. We observed that both larvae and adults show lesser food intake in a dose-dependent manner as compared to their age matched control (Fig. 3A and B).

Impact of shorter and longer ingestion of AgNPs on rate of food intake in F1 adults was evaluated by aging them for different days i.e. day 2, 6 and 14 followed by feeding with a mixture of yeast and blue dye #1 (2.5%). After feeding, foregut and midgut of these flies were dissected and homogenized. The homogenate was centrifuged at 13,500 rpm for 10 minutes and absorbance was recorded at 625 nm using spectrophotometer to quantify the amount of blue dye as a measure of food intake.

We also observed that the adults eclosed from the larvae fed with the food-supplemented with the higher dose of AgNPs show underdeveloped crop and intestine (Fig. 3C). 14 days old flies eclosing from larvae fed with food-supplemented with higher dose of AgNPs (25 mg/L) were eating significantly lesser food than age matched control (Fig. 3D). These results indicate that lesser food intake at larval and adult stages due to ingestion of higher dose AgNPs could also be contributing towards reduced size, weight and development of crop and intestine.

### Alteration in lipid level and accumulation by AgNPs ingestion

**Figure 4:**
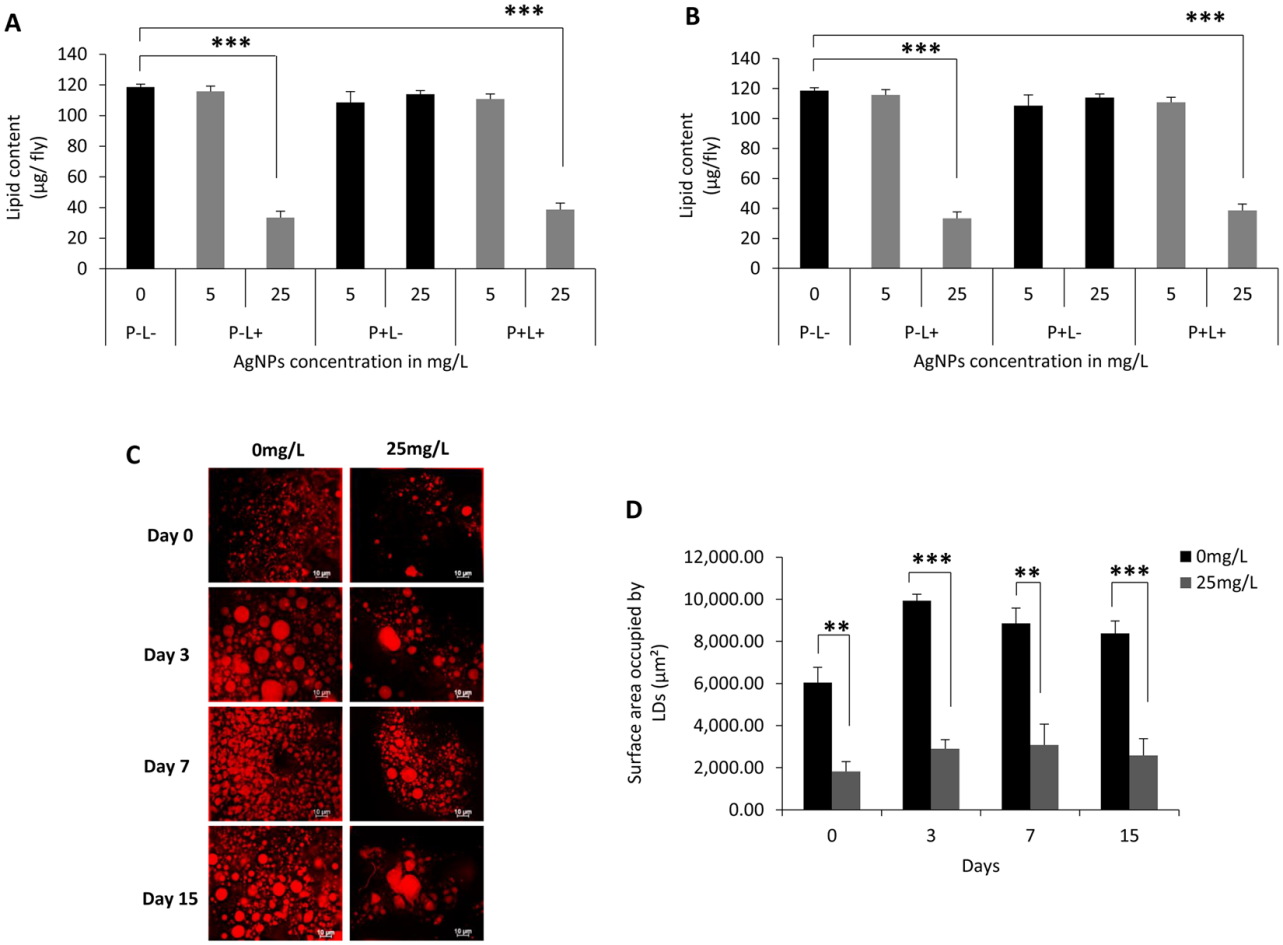
Alteration in lipid levels and accumulation by larval AgNPs ingestion. A notable reduction in lipid content of F1 adult flies on (**A**) Day 0 and (**B**) Day 15 post eclosion. (**C**) Lipid droplets in fat body of control and AgNPs ingested flies. (**D**) Total surface area occupied by lipid droplets. Significance was calculated by using an analysis of variance (ANOVA) followed by Tukey-Kramer MSD post hoc test (MSD α_0.05_: Lipid content (Day 0) = 54.7; Lipid content (Day 15) = 45; LD = 7862.01). ***p<0.001 (Student’s t-test).

In order to understand if the compromised behavioral activities, poor body growth and reduced body weight of adult flies eclosing from the larvae fed with AgNPs-supplemented food is only due to lesser food intake or alteration in metabolic activity is also involved, we investigated lipid levels of these flies. The adult flies eclosing from different ingestion conditions (P − L−, P-L+, P + L− and P + L+) were aged on standard food for up to15 days and their lipid levels were quantified at different time points (day 0, day 3, day 7 and day 15). We observed a dose-dependent reduction in lipid levels of F1 flies that eclosed from larvae fed with AgNPs-supplemented food i.e. P − L+ and P + L+. Interestingly, the flies that eclosed from larvae reared on normal food but their parents ingested AgNPs (P + L−) did not show any alteration in lipid profile (Fig. 4A). Further, aging these small-sized flies on standard food without AgNPs even up to 15 days did not help them recovering their reduced lipid levels (Fig. 4B). The data for F1 flies aged on standard food for 3 and 7 days is not shown as it was comparable to that of 15 days old flies.

Since, a drastic reduction in the lipid profile was observed in these small-sized flies; we dissected their abdominal fat body and stained with nile red dye in order to get insight for sub-cellular localization of the lipid. *Drosophila* fat body is composed of lipid droplets (storage organelle) which is similar to mammalian adipocytes and can be detected by lipophilic nile red stain^32^. We observed a considerably lesser number of lipid droplets in flies reared on higher dose of AgNPs-supplemented food as compared to their age matched controls (Fig. 4C). Quantification of the total surface area occupied by these lipid droplets further confirmed a notable reduction in accumulation of the lipid in lipid droplets (Fig. 4D). Thus, taken together, these results indicate that higher dosage of AgNPs ingestion at larval stage causes considerable reduction in the lipid levels and their accumulation in the fat body.

### AgNPs ingestion affects carbohydrate and protein levels

**Figure 5:**
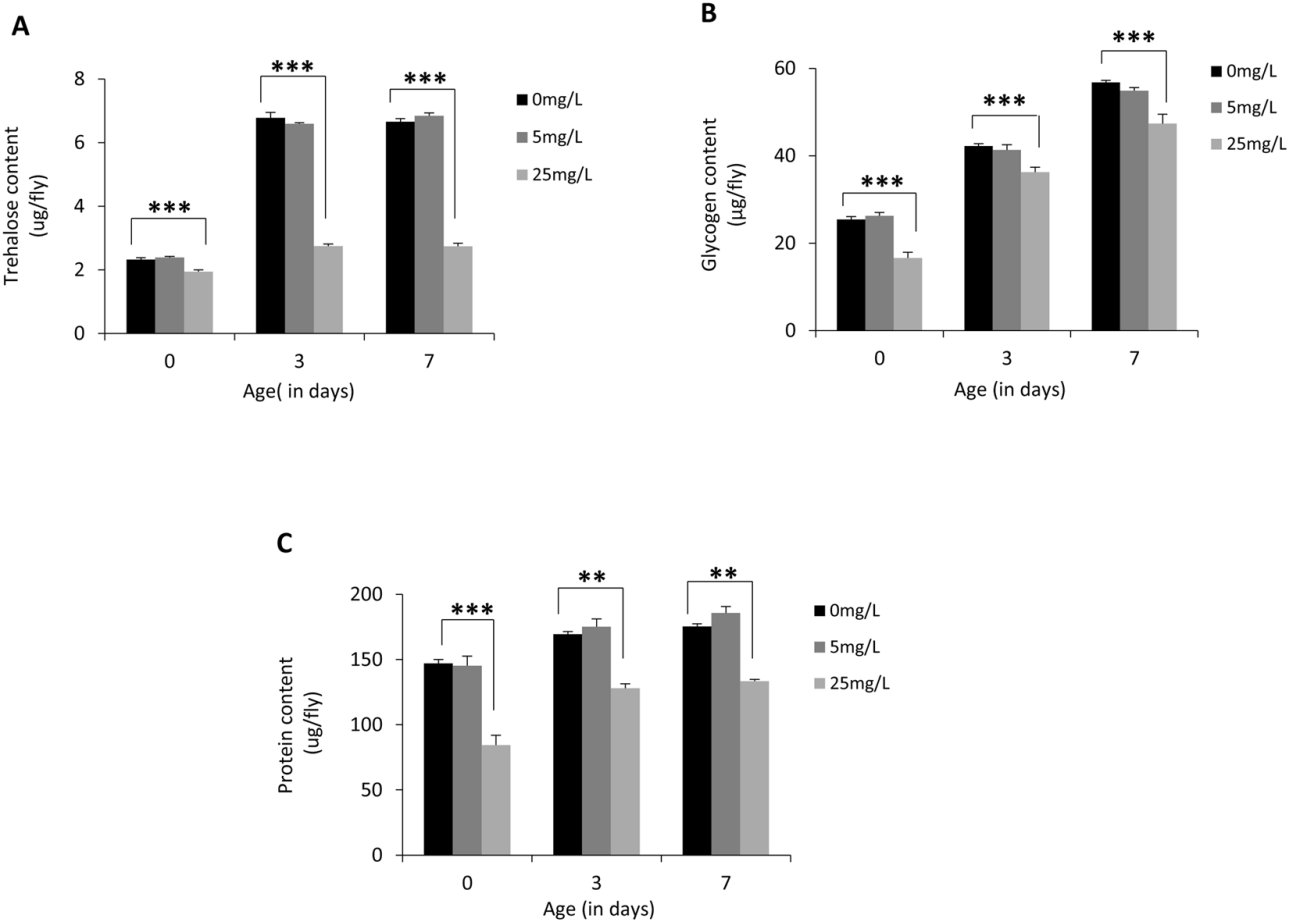
Modulation in trehalose, glycogen and protein content by AgNPs ingestion. AgNPs ingestion in larvae alters (**A**) trehalose content (**B**) glycogen content and (**C**) protein content in F1 adult flies. Significance was calculated by using an analysis of variance (ANOVA) followed by Tukey-Kramer MSD post hoc test (MSD α_0.05_: Trehalose content = 1.0; Glycogen content = 17.5; Protein content = 41.4). ***p < 0.001 (Student’s t-test).

To evaluate effect of AgNPs ingestion on other key energy reservoirs of the body, we estimated trehalose, glycogen and protein in flies eclosed from the larvae reared on control and AgNPs-supplemented food. These flies were aged on standard food for different time points post-eclosion (day 0, day 3 and day 7). Quantitation of energy moieties in these flies displayed reduced levels of trehalose, glycogen and protein only at a higher dosage of AgNPs (25 mg/L) as compared to age matched control (Fig. 5A, B and C). It was interesting to observe that aging of the flies on standard food could not restore reduced carbohydrates and protein levels.

### Increased ROS level by AgNPs ingestion

**Figure 6:**
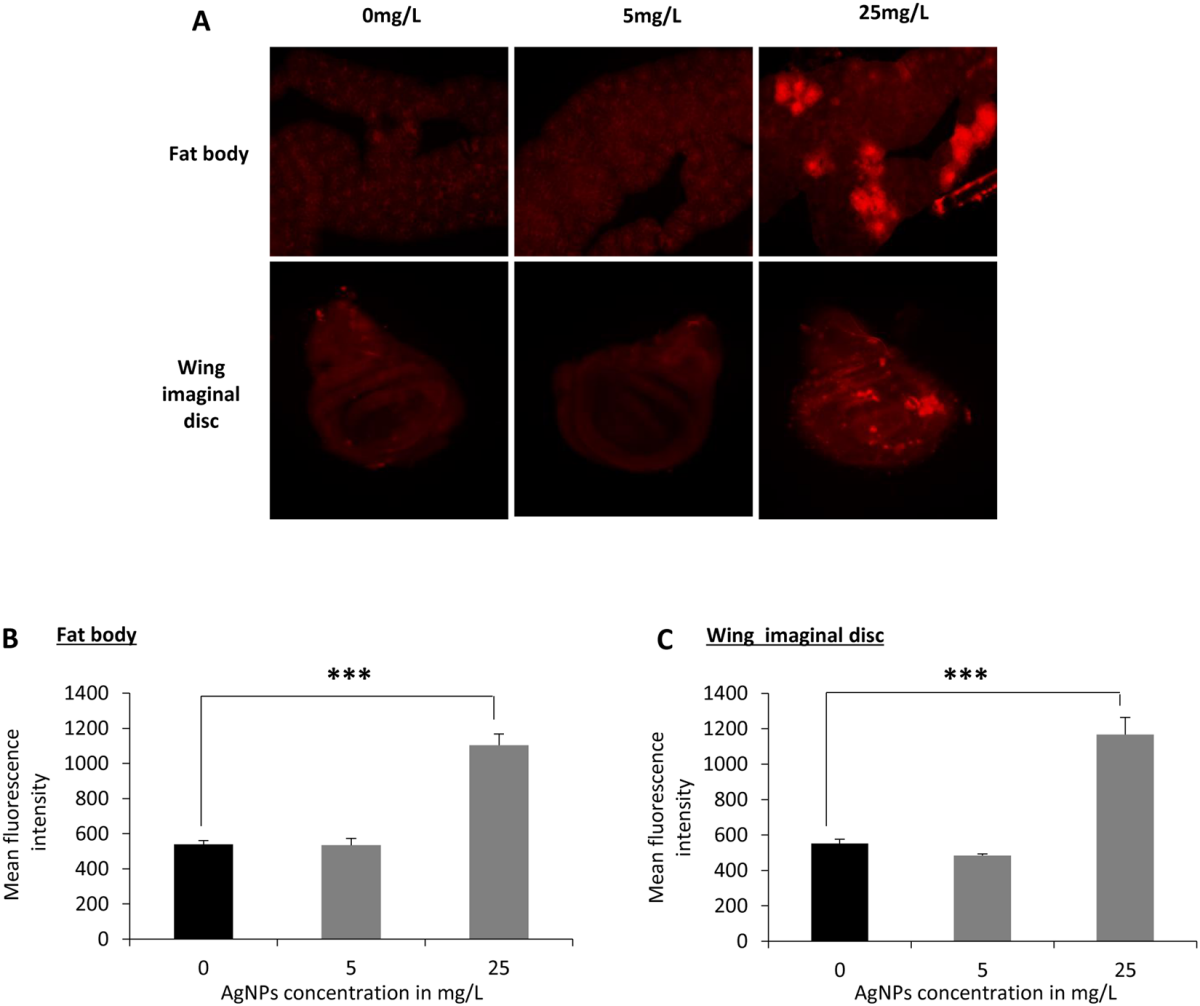
Higher dosage of AgNPs larval ingestion enhanced ROS production. (**A**) ROS production in larval fat body and wing imaginal disc. Quantification of the mean fluorescence intensity of DHE staining in (**B**) larval fat body and (**C**) wing imaginal disc. Significance was calculated by using an analysis of variance (ANOVA) followed by Tukey-Kramer MSD post hoc test. (MSD α_0.05_: Intensity (fat body) = 887.5; Intensity (wing imaginal disc = 757.9). ***p < 0.001 (Student’s t-test).

AgNPs have been reported to cause disruption of mitochondrial respiratory chain and thereby eliciting oxidative stress^33–39^. Therefore, in an attempt to unravel the possible mechanism underlying the alteration in lipid, carbohydrates and protein in progeny eclosed from AgNPs treated larvae, we quantified ROS level in different experimental conditions. The larvae reared on food-supplemented without and with different dosage of AgNPs (0 mg/L, 5 mg/L and 25 mg/L) were observed for ROS levels using DHE in tissues such as fat body, a dynamic tissue involved in multiple metabolic functions. We also monitored ROS in wing imaginal disc to evaluate if tissue other than the one involved in metabolic activity also displays increase in ROS production by AgNPs ingestion (Fig. 6A). The cells producing higher levels of ROS displayed red fluorescent product (ethidium) that intercalates with DNA^40,41^. Our results displayed a significant increase in ROS production in larval tissue (fat body and wing imaginal disc) at a higher dosage of AgNPs as compared to the control (Fig. 6B and C).

## Discussion

In view of the tremendous increase in AgNPs-based consumer products, the potential impact of their frequent exposure to human remains a major concern. Therefore, in order to investigate the effect of AgNPs on behavior and energetics, a detailed and systematic *in vivo* study was performed using *Drosophila melanogaster* as a model system. *Drosophila* serves as an ideal model system for various behavioral studies as some of the integral behavioral aspects in *Drosophila* such as crawling in larvae; climbing and flying ability in adults can be easily monitored^42,43^. Apart from visible behavioral changes, frequent usage of AgNPs containing consumer goods might affect metabolic activity and lead to lack of energy. The metabolic activity of *Drosophila* was determined by their body weight and essential metabolic components of the body such as lipid, carbohydrate and protein levels^44–46^.

As a necessary prerequisite to study impact of AgNPs ingestion, physico-chemical characterization of commercially purchased AgNPs was carried out to ensure their size, shape, level of agglomeration and stability^26^. To elucidate the dosage effect of AgNPs ingestion on behavioral aspect at different developmental stages, adult feeding (parental) and larval feeding (F1) was performed. In the present study, various behavioral assays such as crawling in larvae and climbing of adult flies from parental and F1 generation was monitored. We found that AgNPs administration to adults even for the prolonged period upto 30 days did not affect their climbing ability, however, administration at early developmental stage i.e. larval stage causes impairment of crawling and vertical climbing behavior in a dose-dependent manner^17,18^. We have also reported previously that the integral behavior of the flies i.e. their flying ability gets compromised by ingestion of higher concentrations of AgNPs^31^. The observed loss in flying ability caused by intake of AgNPs suggests that these NPs might disrupt flight by interfering with the pathways responsible for flight or intervention in the functioning of synaptic activity^47–49^ or flight muscles^50^.

As reported earlier^17^, we also observed the characteristic weight loss post eclosion that could not be recovered even when the flies were aged on standard *Drosophila* food for upto 15 days. Moreover, from feeding behavior analysis in control and flies eclosing from the larvae reared on AgNPs-supplemented food, significant loss in body weight by AgNPs ingestion can be attributed to reduced rate of food intake of the F1 larvae. These results suggest that in an attempt to abate the ingestion of AgNPs-supplemented food, *Drosophila* larvae preferred to poor nutrition by minimizing their rate of food intake^51–53^. This compromised feeding behavior in AgNPs fed larvae exerted negative impact on the body size and weight of the adult progeny.

Further, to ascertain that the significant loss in weight and impairment in behavior could be related to energy levels in flies ingesting AgNPs-supplemented food, quantitation of key energy reserves such as lipid, carbohydrate and protein was conducted. Interestingly, we found a significant decline in the level of energy reserves that correlates well with decrease in body weight of AgNPs ingested flies. Additionally, reduction in lipid droplet number in abdominal fat body confirmed our observation that AgNPs ingestion results in reduction in lipid level and its accumulation.

Further, in an attempt to understand the mechanism underlying AgNPs mediated impaired behavior and metabolic activity, a significant increase in the level of ROS positive cells in fat body and wing imaginal discs of the larvae fed with higher concentration of AgNPs-supplemented food was monitored. Cellular exposure to AgNPs has been demonstrated to elicit stress which is indicated by disruption of mitochondrial respiratory chain, increased cytokine and pro-inflammatory mediator release, altered membrane potential and increased ROS^33–39,53–55^. Till date, very limited *in vivo* studies have testified AgNPs to have the potential of causing oxidative stress^35,56,57^. Our findings suggest that the possible reason for abnormal behavior and poor energetics caused by AgNPs ingestion during early larval stage could be generation of ROS.

Taken together, these findings strongly imply that ingestion of higher dose of AgNPs at early developmental stage could exert negative impact on behavior and metabolic activity and therefore, draws an attention towards usage of AgNPs in consumer goods.

## Conclusions

Our study highlights a very important piece of information regarding the possible impact of AgNPs ingestion on behavior and energetics. We demonstrated that dietary intake of AgNPs at larval stage causes behavioral dysfunction by compromising crawling in larvae and climbing ability in adults. Moreover, larval administration of higher dose of AgNPs significantly impaired essential metabolic components of the body that includes lipid, carbohydrate and protein levels in adult flies. Interestingly, the reduced lipid levels was accompanied by minimized accumulation of lipid droplets in *Drosophila* fat body which further established alteration in energetics of flies reared on AgNPs supplemented food. We also observed increased ROS production in AgNPs treated larval tissues which probably is one of the causative factors in metabolic alterations in adult progeny. These findings imply that higher dose of AgNPs ingestion early during development can lead to impairment of behavior along with variation in the energetics in *Drosophila*.

## Methods

### Fly strain, culture and AgNPs treatment

*D. melanogaster* (*Oregon-R*) flies were reared at 25 ± 1 °C on media containing maize flour, yeast, sugar, agar-agar and propionic acid^58^. Fly stock used in the present study were obtained from the Bloomington *Drosophila* Stock Center (BDSC) at Indiana University, Bloomington, USA.

AgNPs used in the present study were obtained from Sun Innovations Corp., Fremont, CA (item #SN1101). An extensive physico-chemical characterization of these commercially purchased AgNPs was conducted and reported earlier^26^. The physical characteristics of AgNPs were defined by using Dynamic light scattering (DLS), Transmission electron microscope (TEM), X-ray diffraction (XRD) and zeta potential measurements for determining their size, shape, purity and level of agglomeration. Prior to adding AgNPs to partially cooled fly food, a stock of 5% (w/v) AgNPs suspension was sonicated (Q_SONICA_ Sonicators) in distilled water to get a homogenous dispersion^26^. Thereafter, the final concentration of 0, 5, 25 and 50 mg/L AgNPs were obtained by mixing AgNPs into the fly food. For the experiments, *Drosophila* food-supplemented without (control) and with different concentrations of AgNPs was used to evaluate its dosage effect.

To investigate the effect of AgNPs on behavior and metabolism at different developmental stages of *Drosophila*, freshly eclosed female and male flies were collected and aged separately on without (control) and with different doses of AgNPs-supplemented *Drosophila* food for 10 days. These females and males were mated two days prior to egg collection and their eggs (F1 progeny) were transferred into the vials containing food without and with different doses of AgNPs. Thus, four different conditions were given to the progeny hatching from the eggs laid by these parents. These conditions are referred as P − L− (parents not fed with AgNPs; F1 larvae not fed with AgNPs), P − L+ (parents not fed with AgNPs; F1 larvae fed with AgNPs), P + L− (parents fed with AgNPs; F1 larvae not fed with AgNPs), P + L+ (parents fed with AgNPs; F1 larvae fed with AgNPs) in subsequent text^30^. It is important to note that ingestion of a dose higher than 50 mg/L had toxic effect on the growth and survival of F1 larvae^25^. Therefore, we could perform all the behavioral assays of F1 progeny at a dose of 50 mg/L of AgNPs that also displayed reduced eclosion rate. For metabolic activity a large sample size was required hence, highest dose of 25 mg/L of AgNPs used.

### Crawling assay

The locomotor activity during early stage of development i.e. wandering third instar larvae from all the above mentioned conditions was recorded by allowing them to crawl in a petriplate containing 3.3% agar-agar with four grooves of same width and depth. For each food condition, the distance covered by each larva placed in a groove was recorded for 30 seconds. Ten larvae per condition were monitored for their crawling ability in the replicates of two.

### Climbing assay

After aging adult flies without and with AgNPs for 10, 20 and 30 days, their vertical climbing ability was monitored using a glass tube. A total of 10 flies were evaluated in two replicate vials per treatment for their climbing ability. Out of 10 flies, the total number of flies that could climb 10 centimeters in 15 seconds in three repeats per vial was recorded. Similarly, the F1 progeny that was fed on food-supplemented without and with AgNPs throughout development were collected on day 0 post-eclosion and their climbing ability was also recorded on day 1 and 7 post-eclosion.

### Feeding assay

The rate of food intake at larval and adult stage was determined by feeding them with a mixture containing yeast paste and blue dye (FD & C Blue dye #1, Sigma Aldrich). For larval food intake estimation, a hole (2.5 mm diameter) was created in the center of a petri dish containing 3.3% (w/v) agar-agar and filled with a mixture of yeast paste with 4% blue dye #1. Thereafter, a total of 10 third instar larvae from each treatment condition were transferred into the hole and allowed to feed on yeast-dye mixture for 2 hours in the replicates of two. After feeding, larvae were washed with ice-cold water, dried and homogenized in 200 ul of 1X PBS. The homogenate was centrifuged at 35,000 rpm for 10 minutes and absorbance for blue dye at 625 nm was read using spectrophotometer (EC Microscan, M55605A).

For estimation of adult food intake, flies aged for different time points were collected in the group of 10 from each condition and allowed to feed on a mixture containing yeast and 2.5% blue dye for 30 minutes at 25 °C. After feeding, the region between crop and midgut of alimentary canal was dissected and homogenized. The homogenate was centrifuged at 13,500 rpm for 10 minutes and absorbance was recorded at 625 nm using a spectrophotometer (EC Microscan, M55605A). Three replicates per condition (control and AgNPs treated) were used for the assay.

### Glycogen estimation

A total of 4 flies in the replicate of five were homogenized in 400 µl of 2% Na_2_SO_4_. 20 µl of the homogenate was aliquoted and mixed with 46 µl of 2% Na_2_SO_4_ and 934 µl of chloroform/methanol (1:1). The mixture was centrifuged at 13,500 rpm for 10 minutes, the supernatant was discarded and pellet containing glycogen was air dried for 10 minutes. The anthrone reaction of the pellet was carried out by adding 500 µl of anthrone reagent (0.2% anthrone in 72% sulphuric acid) in centrifuge tubes containing pellet and vortexed. The mixture was heated at 90 °C for 20 minutes and vortexed at an interval of 5 minutes. The color of the reaction mixture changes from yellow to green on heating. The tubes were cooled on ice for 10 minutes and then returned to room temperature for 20 minutes. The absorbance was noted at 620 nm. The glycogen concentration was calculated using a D-glucose standard curve.

### Trehalose quantification

4 flies were homogenized in 500 μl of 70% ethanol and the pellet obtained was air dried. Pellets were re-suspended in 200 μl of 2% NaOH, heated at 100 °C for 10 minutes and cooled on ice. Anthrone reaction was carried out by adding 750 μl anthrone reagent to 100 μl of the sample. The absorbance of supernatant was recorded at 620 nm using spectrophotometer (EC Microscan, M55605A). Five replicates per condition were used for trehalose measurement.

### Protein quantification

4 flies in one replicate were homogenized in 400 µl of 2% Na_2_SO_4_ and 0.05% Tween 20 (1:1). 80 µl of the homogenate was aliquoted in fresh tubes and 500 µl of 0.15% sodium deoxycholate was added. The mixture was kept on ice for 10 minutes and 1 ml of 3 M trichloroacetic acid was added. The tubes were spun at 8,500 rpm for 15 minutes at 4 °C. After discarding the supernatant, pellet was rinsed only once with 1 ml of 1 M HCL, air dried and dissolved in 1.6 ml of bicinchoninic acid (BCA) working reagent. The mixture was heated at 60°C for 10 minutes and tubes were then kept on ice to stop further color development. The absorbance was recorded at 562 nm using spectrophotometer (EC Microscan, M55605A). Experiment was performed with 5 replicates.

### Lipid estimation

A group of 10 freshly emerged flies were over etherized and weighed immediately using Citizen CM11 microbalance to obtain fresh weight. Thereafter, the samples were dried at 70 °C for 36 hours and weighed again to obtain dry weight. The difference between fresh and dry weight is indicative of water content in the fly. After drying, the ether soluble lipids were extracted from the sample by adding 1 ml di-ethyl ether to dried flies stored in 1.5 ml microcentrifuge tube. These tubes were kept on shaker for 48 hours with three ether changes at an interval of 12 hours. After the last ether change, flies were dried for 2 hours at 30–35°C and weighed to obtain lipid free weight of flies. The difference between dry weight and lipid free weight was considered as the total lipid content of the flies. Five replicate vials per condition were set for this assay.

### Lipid droplet staining

For lipid droplet staining in adult fat body, the abdomen of age-matched flies was dissected in ice-cold 1X PBS fixed in 4% formaldehyde for 20 minutes. After fixation, the samples were washed thrice in 1X PBS. The sheet of fat body was taken out from dorsal surface of abdomen and stained with freshly prepared 1:2000 dilution of 0.5 mg/ml nile red (Sigma Aldrich- N3013) in PBS for 30 minutes. After staining, tissue was rinsed twice with 1X PBS and mounted in Vectashield mounting medium. Samples were examined under Nikon Eclipse (Ni-E) fluorescence microscope and lipid droplet quantification was performed using NIS-Elements AR software. Minimum five samples per condition were analyzed for lipid droplet analysis and quantification.

### Reactive oxygen species (ROS) detection and imaging

Dihydroethidium (DHE) staining was performed to monitor superoxide radicals^3,11,59^ in the fat body and wing imaginal disc of third instar larvae. To evaluate ROS production in *Drosophila melanogaster*, larvae were reared on food without and with different concentrations of AgNPs. A stock of 30 mM solution of DHE, (Invitrogen Molecular Probes, cat no. D11347) was prepared just before use by reconstituting in anhydrous dimethyl sulfoxide (DMSO; Sigma-Aldrich, cat. no. 276855). To allow optimal respiration, the tissue of interest was dissected from these larvae in 1X Schneider’s medium (+) L-glutamine (GIBCO, cat no. 21720024). The reconstituted dye solution (30 mM) was diluted in 1X Schneider’s *Drosophila* medium to obtain the working solution (50 µM) and vortexed to disperse the dye. The tissue was incubated with the dye in dark chamber for 4 minutes soon after dissection at room temperature. This was followed by rinsing the tissue with 1X Schneider’s medium and imaging using Nikon Eclipse (Ni-E) fluorescence microscope. ROS quantification was performed using NIS-Elements AR software.

### Statistics

Statistical analysis was performed using analysis of variance (ANOVA) in IBM SPSS statistical package (version 22) for each experiment conducted. The difference between control and different concentrations of AgNPs were compared by Tukey–Kramer minimum significant difference post hoc test (MSDα0.05). Statistical significance in each graph is shown by Student’s t-test.
